# Does HIV Exploit the Inflammatory Milieu of the Male Genital Tract for Successful Infection?

**DOI:** 10.3389/fimmu.2016.00245

**Published:** 2016-06-24

**Authors:** Rachel T. Esra, Abraham J. Olivier, Jo-Ann S. Passmore, Heather B. Jaspan, Rushil Harryparsad, Clive M. Gray

**Affiliations:** ^1^Department of Pathology, Division of Immunology, Faculty of Health Sciences, Institute of Infectious Disease and Molecular Medicine, University of Cape Town, Cape Town, South Africa; ^2^Department of Pathology, Division of Virology, Faculty of Health Sciences, Institute of Infectious Disease and Molecular Medicine, University of Cape Town, Cape Town, South Africa; ^3^National Health Laboratory Services, Cape Town, South Africa

**Keywords:** HIV-1, medical male circumcision, sexually transmitted infections, inflammation, risk factors, acquisition, exploitation

## Abstract

In many parts of the World, medical male circumcision (MMC) is used as standard prevention of care against HIV infection. This is based on seminal reports made over 10 years ago that removal of the foreskin provides up to 60% protection against HIV infection in males and seems currently the best antiretroviral-free prevention strategy yet against the global epidemic. We explore the potential mechanisms by which MMC protects against HIV-1 acquisition and that one of the oldest, albeit re-invented, rituals of removing a foreskin underscores the exploitative nature of HIV on the anatomy and tissue of the uncircumcised penis. Furthermore, foreskin removal also reveals how males acquire HIV, and in reality, the underlying mechanisms of MMC are not known. We argue that the normal sequelae of inflammation in the male genital tract (MGT) for protection from sexually transmitted infections (STI)-induced pathology represents a perfect immune and microbial ecosystem for HIV acquisition. The accumulation of HIV-1 target cells in foreskin tissue and within the urethra in response to STIs, both during and after resolution of infection, suggests that acquisition of HIV-1, through sexual contact, makes use of the natural immune milieu of the MGT. Understanding immunity in the MGT, the movement of HIV-1 target cells to the urethra and foreskin tissue upon encounter with microbial signals would provide more insight into viral acquisition and lay the foundation for further prevention strategies in males that would be critical to curb the epidemic in all sexual partners at risk of infection. The global female-centric focus of HIV-1 transmission and acquisition research has tended to leave gaps in our knowledge of what determines HIV-1 acquisition in men and such understanding would provide a more balanced and complete view of viral acquisition.

## Introduction

There are approximately 35 million people living with HIV globally with Sub-Saharan Africa (SSA) accounting for more than two-thirds of global HIV/AIDS infections (1). Adolescent girls and young women are disproportionately affected by HIV in SSA, and 58% of the population living with HIV are estimated to be women (1). The high prevalence of HIV acquisition in females is most likely to be multifactorial, associated with the presence of STIs, genital inflammation, and exogenous hormone contraceptive use (2, 3). There are also sociodemographic factors that may underlie higher prevalence in women, such as age discrepancies in relationships, gender-power imbalances, and gender-based violence that contribute the higher rates of infection in young women (2, 3). What is perhaps missing in these equations is how males acquire HIV, as male-to-female transmission is likely the source of transmission and a driver behind the high rates of infection measured in women. Men who have sex with men (MSM) are 19 times more likely to be living with HIV than the general population (4) and the prevalence of HIV in MSM is rising in several parts of the world (5). Little is known about the biological determinants of HIV transmission in male-to-male, male-to-female, or female-to-male sexual networks underlying the high prevalence of HIV in high-risk groups such as adolescents in SSA and MSM. One other possible determinant, perhaps overlooked, is that a high proportion MSM in Africa also report recent female sexual partners (6). HIV genotype studies at the early stages of the epidemic in South Africa showed that subtype B HIV-1 was confined mostly to the MSM community while subtype C circulated mostly in the heterosexual population, suggesting two separate HIV-1 introductions into this region (7). More recent studies in South Africa, Kenya and Senegal show that this has changed with MSM predominately being infected with the same variants as the heterosexual population (8–10). Conceivably, this might suggest a homogenization of epidemics between heterosexual and MSM sexual networks in the regions with the highest global prevalence of HIV (8, 10). Although other reviews focus on factors that influence female risks of HIV acquisition, few have focused on possible determinants of risk factors in males, which likely provides a niche in the male genital tract (MGT) conducive for HIV acquisition.

The low estimates of HIV transmission risk per sex act between males and females do little to explain the magnitude of the HIV epidemic, both globally and in SSA. Recent meta-analyses have reported the risk of heterosexual HIV acquisition in developed countries (expressed per 10,000 sexual exposures) to be four for insertive and eight for receptive penile–vaginal intercourse (11). The same analysis reports a significantly higher risk estimate for the MSM population with the risk of HIV acquisition to be 138 for receptive and 11 for insertive anal intercourse per 10,000 exposures (11). Estimates of the risk of HIV acquisition in SSA are difficult to accurately determine due to substantial heterogeneity in published data. Meta analyses from 14 sites in eastern and southern Africa have estimated that both men and women are at a higher risk of HIV acquisition through heterosexual contact when compared to data in developed countries: 10 and 9 per 10,000 exposures, respectively (12). These estimates raise relevant and important questions. Why is the magnitude of the HIV epidemic so high in SSA? Are there cofactors, both biological and/or social, that might explain the high rates of global heterosexual acquisition of HIV? HIV acquisition risk is unlikely to be defined by a “one size fits all” transmission probability; where age, other sexually transmitted infections (STIs), circumcision status, disease stage, antiretroviral (ARV) use, viral load, and viral subtype, all contribute to the efficiency of viral transmission (13).

There is no doubt that current research on HIV acquisition and prevention in females is of utmost importance, but this focus leaves large gaps of knowledge in the determinants of HIV acquisition, and hence transmission, in males. We know that medical male circumcision (MMC) is 56–61% effective at preventing HIV acquisition in men (13–15), but we do not fully understand how circumcision protects against HIV and other STIs at a structural, cellular, and molecular level. That such an ancient ritual of circumcision, albeit modernized, can have such a profound effect on preventing viral acquisition in males underscores the exploitative nature of HIV on the anatomy and tissue of the uncircumcised penis. How does HIV use the MGT environment and anatomical structures to promote its acquisition in men?

## Foreskin Barrier Integrity and Immunity

**Figure 1:**
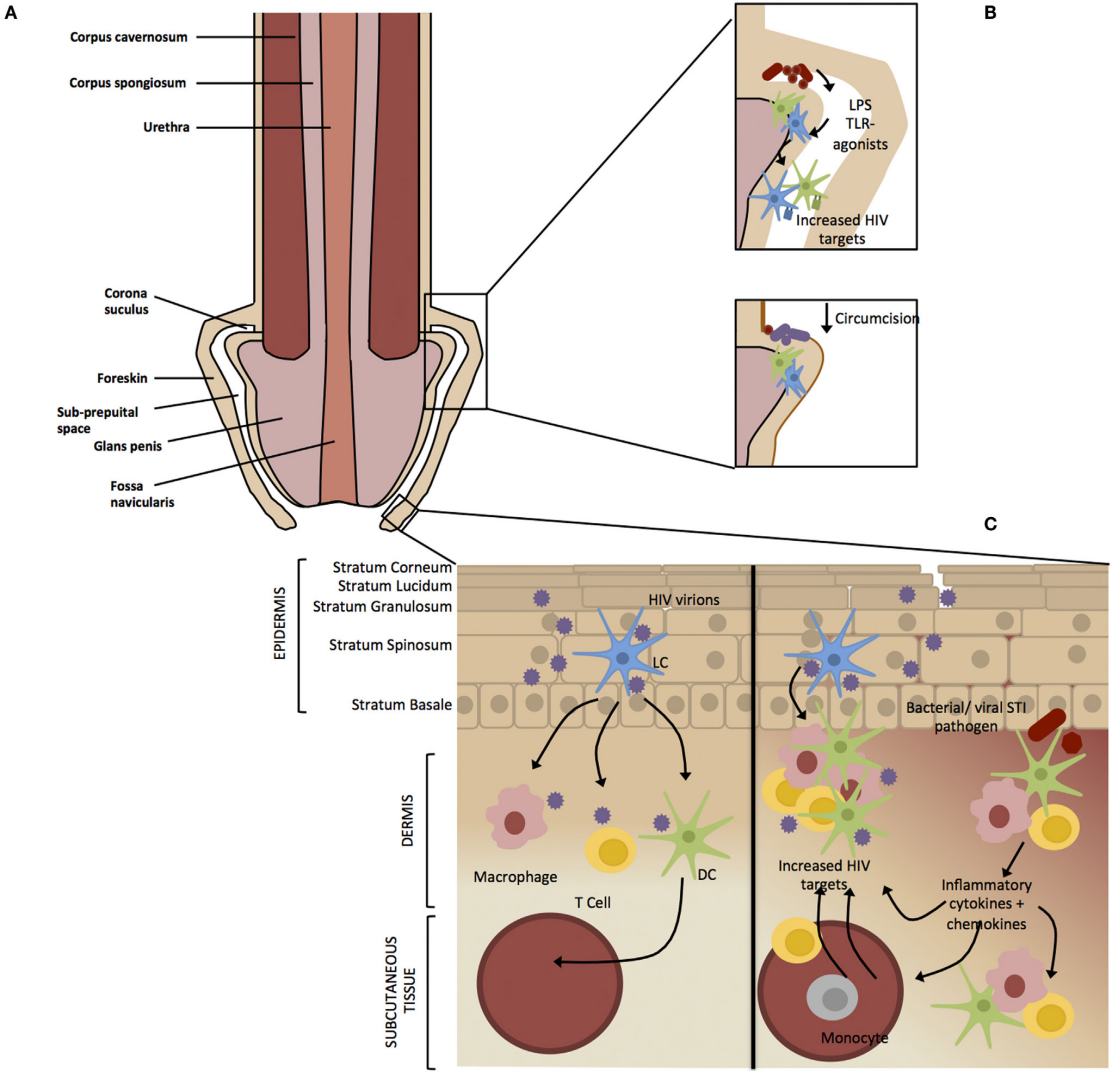
**Factors affecting HIV infection of the male genital tract**. **(A)** The foreskin consists of a double layer of stratified epithelium that covers the glans/corona and meatus of a flaccid penis. **(B)** Circumcision results in the removal of the majority of the foreskin epithelium leaving a “dry” keratinized surface that is assumed to be resistant to HIV infection. Non-STI genital microbial populations have been shown to modulate genital inflammation through the antigen recognition, which may result in migration or activation of HIV target cells into the foreskin. Circumcision results in a removal of the moist sub-preputial space and decrease in anaerobic bacterial species, which are likely pro-inflammatory. **(C)** The foreskin is a stratified epithelium consisting of six epidermal layers namely stratum corneum (SC), stratum granulosum (SG), stratum spinosum (SS), and stratum basale (SB). HIV virions crosses the keratinized (light brown) epithelial border through micro abrasions that occur during sexual intercourse or through the formation of viral synapses between HIV-infected cells and epithelial cells (20). Langerhans’ Cells (LCs) reside within the epidermis where they are the first to encounter the virus (21). Activated LCs migrate into the lower dermal tissues, transferring the virus to resident dermal immune cells such as T cells, macrophages, and epithelial dendritic cells (DCs). Dendritic cells act as professional antigen presenting cells, phagocytosing virions, and migrating to the draining lymph nodes where they present HIV antigens to immature T cells and B cells (22). Intact virus can be trafficked into the lymph nodes by DCs and transferred to CD4^+^ T cells (23) where the virus replicates and is disseminated throughout the body (24). Asymptomatic STIs do not present clinically but inflammation still occurs on a cellular level. The release of pro-inflammatory cytokines by keratinocytes and dermal immune cells in response to an STI would result in recruitment and activation immune cells to the site of infection. This inflammatory environment would result in an accumulation of HIV target cells and therefore increase the efficiency of viral transmission in the case of an HIV infection.

The protective effect of circumcision against HIV infection indicates that the foreskin is an important entry point of HIV in the MGT. The MGT consists of the penile urethra and the testes and comprises both simple and stratified epithelia (16). Although not necessary for normal penile functioning, the foreskin is thought to confer physical and immunological protection to the sensitive glans penis (Figure 1A) (17). The foreskin is a common site of entry for other STIs and is susceptible to micro-abrasions during intercourse, which likely leads to inflammation and resident HIV target cell exposure making it an important immunological area of the MGT (18). In the same way that HIV has evolved to exploit lymphoid structures to efficiently propagate systemically in the body (19), HIV utilizes the structures in the foreskin and urethra, along with the underlying immune cells, as a portal into lymphoid structures and systemic circulation.

The foreskin consists of a double layer of stratified epithelium containing squamous keratinocytes that covers the glans/corona and meatus of a flaccid penis (18). It believed that the removal of the majority of the foreskin epithelium through circumcision results in a “dry” keratinized epithelium of the glans/corona that is though to be more resistant to microabrasions during sexual intercourse and limiting the chances for HIV to contact target cells residing in dermis and epidermis, compared the “wet” mucosal epithelium of the glans/corona covered by the foreskin in uncircumcised men (25). The keratin layer is believed to provide the first line of innate defence against infection of the penile tissue although published data differs with regards to the degree of foreskin keratinization, and the difference between keratin thickness of the inner and outer foreskin tissues (26). In an uncircumcised penis, the foreskin retracts during erection, exposing the glans penis as well as the inner foreskin, which is thought to be more susceptible to viral acquisition compared to other penile tissues (27). This is based on observations from explant studies that have shown the inner foreskin to be exhibit higher inflammatory cytokine responses and HIV target cell activation compared to the outer foreskin, leading to the hypothesis that HIV target cells in the inner foreskin have increased interaction with external factors (27, 28). During sexual intercourse, the inner foreskin is exposed to vaginal and/or rectal secretions and is susceptible to micro-abrasions, possibly creating additional portals for HIV entry.

The foreskin was shown to contain a high density of the HIV target cells (29, 30), and a higher density of CD4^+^ T cells and langerin expressing Langerhans’ cells (LCs) were identified in the inner compared to the outer foreskin (30, 31). Prodger et al. described an “inflammatory immune microenvironment” within the foreskin where they observed an upregulation of HIV co-receptors on CD4^+^ T cells as well as increased production of inflammatory cytokines, TNF-α, and IFN-γ by CD8^+^ T cells (32). A similar observation was made by Lemos et al., suggesting that the protective effects of circumcision may be due to the removal of inflamed tissue that is not only more permeable to HIV but would possess a higher density of HIV target cells (33).

Stratified epithelium, typically found on surfaces exposed to the environment, consists of multiple layers of epithelial cells forming a physical barrier against external pathogens (34). High numbers of immune cells, such as LCs, which serve as specialized antigen presenting cells, are typically present in stratified epithelium (35). LCs have been identified in the upper layers of the epidermis and are proposed to be the first cells to come into contact with HIV upon infection (36). Other HIV target cells such as CD4^+^ T cells, dendritic cells (DCs), and macrophages expressing C-type lectins reside in the lower dermis. The gp120 subunit of HIV Env binds the C-type lectin langerin expressed by circulating LCs where after the virus is either internalized and degraded or transferred to a CD4^+^ T cell, depending on viral load (21, 31). DCs express the C-type lectin DC-SIGN, which is also able to bind to gp120 and may promote transfer of HIV-1 from DCs to CD4^+^ T cells (23, 37). Macrophages are able to selectively capture HIV-1-infected CD4^+^ T-cells leading to the infection and efficient transfer of HIV-1 from cell-to-cell (38) These cells are found in surfaces typical of the meatus (distal end of penile urethra), fossa navicularis (opening), and foreskin (39). Keratinized stratified squamous epithelium spans the exterior of the glans penis and foreskin and transitions into non-keratinized stratified squamous epithelium in the fossa navicularis (Figure 1A) (16).

## Urethra: The Mucosal Surface of the MGT

In contrast to the stratified squamous epithelium of the foreskin, a simple layer of non-keratinized pseudo stratified columnar epithelial cells is found along the penile urethra and provides a lower level of protection against HIV infection (Figure 1A) (39). The urethral epithelium has a high density of intraepithelial immune cells including CD8 T cells, natural killer cells, as well as HIV-1 targets CD4 T cells, DCs, and macrophages (16). As the urethra is the primary site of infection for many bacterial and viral STIs, this mucosal surface represents an important component of the penile protective immune tract, bit also implicating it as a portal of entry to HIV and other STI that use these cells for productive infection during or after sexual intercourse (40, 41). Present knowledge of HIV infection through the urethra is incomplete due to the limited availability of fresh urethral samples, although cells expressing HIV-1 co-receptors have been identified at the urethral opening (42). It is thus conceivable that the urethral opening may be an underestimated vulnerable site of HIV infection in circumcised men (25). An *ex vivo* study on penile tissue obtained following gender reassignment surgery found tissue explants from the foreskin, glans, meatus, and urethra to be equally susceptible to R5 HIV-1 infection (43, 44). Another study using penile tissues from cadavers found the inner foreskin to be more susceptible to HIV-1 infection compared to the outer foreskin, glans, shaft and urethral opening (44). Through the use of urethral tissue explants and the development of a novel *in vitro* reconstructed urethral mucosa, Ganor et al. identified the urethra as an entry site for HIV-1 infection, where urethral macrophages, and not T cells or LCs, were the initial HIV-1 target cells at the urethral opening (45). It is known that approximately 40% of men are not protected after MMC, and evidence pointing to the urethra as an HIV-1 portal suggests that further focus should be made at understanding viral acquisition at this unique mucosal surface.

## The Impact of MMC and Other STIs on HIV Risk

Data from MMC trials give strong evidence for the protective effect of circumcision against HIV acquisition (13–15), although the findings were not as conclusive concerning other STIs. The Uganda trial showed that MMC of adolescent boys and men resulted in a 45% overall reduction in genital ulcerative disease (GUD) (46), and both Ugandan and South African trials showed a significant reduction in Herpes Simplex Virus-2 (HSV-2) acquisition (46, 47). Contradictory results emerged from the Kenyan trial indicating no significant decrease in HSV-2 infections as a result of MMC (48), suggesting that the reduction in HIV due to circumcision was independent of HSV-2 and GUD (48). Weiss et al. found that circumcision significantly reduced the risk of chancroid and syphilis but was only weakly associated with a reduction of HSV-2 infection (49). It was suggested that the warm, moist area beneath the foreskin encourages pathogenic growth and that stricter hygiene of the prenuptial space (between the foreskin and glans penis) is associated with lower HIV prevalence. This would indicate that the dynamic environment occupying this space has an effect on HIV risk (50, 51). Thus the removal of such an environmental niche for these pathogens is more protective against diseases that commonly cause lesions on the foreskin tissue, such as cancroid, compared to lesions caused by syphilis and HSV-2, both of which present more widely across the male genitalia (52). Follow up from the Kenyan trial demonstrated that circumcision did not decrease the risk of *Neisseria gonorrhea* (NG), *Chlamydia trachomatis* (CT), and *Trichomonas vaginalis* and is thus not protective against non-ulcerative genital diseases (NUDs) (53). While there has been epidemiological evidence of decreased prevalence of cervical neoplasia if females with circumcised partners (54–56), observational studies have found inconsistent results with regards to impact of circumcision on the transmission of HPV to female partners (57, 58). Results from the Ugandan circumcision trial found a lower incidence of high-risk HPV infection in women with circumcised partners, leading the authors to hypothesize that male circumcision involves a reduction of penile HPV carriage (59). Data on the effect of circumcision on HPV prevalence in men are inconsistent (60), and various studies have reported an increased prevalence of HPV infection in uncircumcised men (61). Hernandez et al. found that circumcision did not increase the risk HPV acquisition, but rather reduced viral clearance for both oncogenic and non-oncogenic forms of the virus (62). The higher prevalence of HPV in uncircumcised men therefore could be attributed to a longer duration of infection rather than increased acquisition (62), highlighting that pathogen-specific factors need to be considered in the epidemiology of STIs in men.

Despite the seeming disparity on the effectiveness of MMC in reducing the risk for other STIs, there is evidence to indicate reductions in the incidence of GUDs following MMC although the biological mechanism through which this would impact HIV risk is unclear. It is biologically plausible and likely that mucosal disruptions as result of ulcerative STIs would provide additional routes of transmission for HIV acquisition in males. In addition, inflammation from STIs could increase the efficiency of HIV infection by recruiting and activating HIV target cells that reside and migrate to the foreskin tissues (33). This inflammatory effect may persist after clearance of the infection as demonstrated by Donoval et al. who found a higher proportion of HIV target cells in the foreskins of men with a history of STIs compared to those who had no STI history (63). Sbazo et al. have also suggested that the reduction of the highly vascularized frenulum, which is susceptible to trauma during sexual intercourse as well as ulcerative lesions, is one of the mechanisms by which circumcision prevents the synergy found between HIV and others STIs (64).

## Are Asymptomatic STIs Elevating HIV Acquisition Risk in Males?

Sexually transmitted infections have been identified as a significant factor increasing the risk of transmission and acquisition of HIV (65, 66), where pre-existing STIs increase the risk of HIV acquisition by two to threefold (67) and that ulcerative STIs are an even higher risk factor than non-ulcerative STIs. These findings led to the hypothesis that STI treatment may be an effective HIV intervention strategy in populations where HIV and other STIs are prevalent. Initial results from a randomized controlled trial (RCT) in Mwanza, Tanzania showing a 38% reduction of HIV incidence as a result of treating STIs (68). However, subsequent data from nine RCTs could not reproduce this finding and there was no significant reduction of HIV incidence when symptomatic STIs were treated (65). These incongruous results have brought into question whether symptomatic STI treatment should be seen as an HIV intervention (69). Such a syndrome-based approach to STI management, employed by multiple countries in SSA, may underestimate the impact of subclinical inflammation due to asymptomatic and non-ulcerative STIs that may contribute to HIV susceptibility. Regardless of visible ulcerations, HSV-2 has been linked to an approximately threefold increased risk of HIV acquisition.

A case for persistent immune activation was observed after successful treatment of HSV-2 with Acyclovir, where increased expression of mucosal CCR5^+^ CD4^+^ T-cells remained at the sight of herpetic ulcers long after infection had been cleared (70). These findings would suggest that an initial immune activation event results in the persistence of HIV target cells in the MGT long after HSV-2 ulcerative resolution. Prodger et al. observed a higher proportion of HIV target CCR5^+^ T cells in the foreskins of Ugandan men with asymptomatic HSV-2 (71), supporting previous findings that HSV-2 infection increases the density of HIV target cells in the foreskin (72). Collectively, these data support the hypothesis that an asymptomatic or cleared HSV-2 infection may increase the susceptibility of the foreskin to HIV infection. In addition to an increased density of HIV target cells, asymptomatic HSV-2 has been associated with decreased expression of the epithelial junction protein Claudin in the foreskin, creating a compromised epithelial barrier that may be more susceptible to HIV-1 infection (73). Experiments using genital epithelial monolayers demonstrated that exposure to HIV-1 directly impairs mucosal barrier integrity through the disruption of tight junction markers, namely ZO-1 and Occulin enhancing HIV viral entry to sub mucosal targets (74). The disruption of the mucosal barrier, as a result of direct HIV exposure or coinfection with another STI, and the resultant epithelial cell induced inflammation (74) may be another mechanism HIV utilizes to gain access to target cells in the genital sub mucosa.

## The Penile Microbiome and Immune Integrity

The link between the microbiome and immunity has clearly been shown in the intestine (75, 76) and the way by which commensal microbiota, and released metabolites, shapes immunity in the skin is a focus of current research (77–79). Circumcision has been shown to alter the diversity and prevalence of the penile microbiota – a potential mechanism explaining the protective effect of circumcision against HIV (80, 81). Non-STI-genital microbial populations have been shown to modulate genital mucosal inflammation through antigen recognition and thus increase HIV risk through the activation of HIV target cells (82, 83). Price et al. showed that the microbiota of the coronal sulcus, the junction between the shaft and glans of the penis, predominately consists of anaerobic and putative vaginal taxa prior to circumcision and that anaerobes and skin taxa dominate following circumcision (81). Similarly distinct microbiota were shown to differentiate the coronal sulcus of circumcised and uncircumcised men (84). There was no difference between the microbial diversity within first pass urine samples, as a measure of urethral microbiome, collected from the same men, before and after circumcision, indicating that circumcision has no impact on the microbial diversity of the urethra (84). The moist sub-preputial space below the foreskin is thought to provide an anoxic microenvironment that harbors anaerobic bacterial species (81, 85). Predominantly anaerobic vaginal microbiota associated with bacterial vaginosis (BV) in women increases inflammation and HIV susceptibility (86, 87) in the female genital tract. Circumcision has been associated with a reduction of BV in female partners (88, 89) as well as a reduction of anaerobic bacterial species colonizing the coronal suculus (81). The pro-inflammatory microbial community of the sub-preputial space may have a knock-on effect on other possible points of HIV entry as the foreskin covers the urethral opening in the majority of uncircumcised men (85). Based on this, it is postulated that the anaerobes on the uncircumcised penis are likewise pro-inflammatory, and thus result in migration or activation of HIV target cells into the foreskin (81), thereby increasing the likelihood of HIV infection through the urethra and foreskin in uncircumcised men (25) (Figure 1B).

## Does HIV-1 Exploit the MGT?

The role of subclinical inflammation in increasing susceptibility of the foreskin to HIV infection is unclear, especially in developing countries where a syndrome-based approach to STI management is followed. A high prevalence of commonly asymptomatic STIs such as CT and NG have been reported in high-risk MSM cohorts (90, 91), although there is little data on the prevalence of asymptomatic STIs in the general population. Asymptomatic STIs in men may be the driver of elevated pro-inflammatory cytokines and chemokines in foreskin tissue and the urethra, thereby recruiting activated HIV target cells to the site of infection (Figure 1C). Endocervical epithelial cells are the predominant niche for initial infection of *Chlamydia*, a primarily asymptomatic STI (92) and is one of the most prevalent STI in both men and women. *Chlamydia* infection leads to an inflammatory cascade associated with an influx of HIV-1 target cells as a result of the release of pro-inflammatory cytokines by epithelial cells (93). Buckner et al. showed that HIV infection of CD4^+^ CCR5^+^ cell lines is enhanced by exposure to supernatant from *Chlamydia*-infected epithelial cells. This infers that *Chlamydia* infection may facilitate viral infection in the local environment (94).

We thus posit that asymptomatic STIs increase the risk of HIV infection through subclinical inflammation that is modulated by epithelial dysbiosis. The inflammatory events in the MGT upon STI infection are a natural sequelae for protective immunity around the penile tissue. However, such subclinical inflammation in the foreskin/urethra of uncircumcised males and the urethra in circumcised men represent a perfect niche for HIV-1 to exploit in establishing a successful productive infection. Similar to the female genital tract and all human mucosal surfaces, innate immune defences such as mucus production, pattern recognition receptors, and antimicrobial peptide production are present in the MGT [Reviewed in Ref. (16)]. There is evidence that penile immunity is quite active in the FS, urethra, and within the epithelial tissues (95) and it would seem intuitive that immune protection within the MGT is a critical survival advantage to the host and therefore used to a survival advantage by HIV-1.

## Conclusion

Medical male circumcision has been widely accepted as an effective intervention strategy for the heterosexual transmission of HIV (96, 97) and has been shown to be a more effective intervention strategy than STI treatments, vaccines, and microbicides (29). Complications as a result of MMC were low after the MMC trials (between 1.7 and 7.6%) and were mostly of minor clinical significance (98, 99). The prevalence of traditional circumcision performed in a non-clinical setting is thought to be between 20 and 80% in Eastern and Southern Africa (54) and has been associated with serious medical complications (100). Although the World Health Organisation reported 9.1 million MMCs performed between 2007 and 2014 in priority countries in East and Southern Africa (97), traditional circumcision in is still prevalent in these regions for reasons that are both cultural and based on health service capacity (100). More research is needed to evaluate the impact of traditional circumcision on HIV transmission rates and well as on the acceptability of MMC in communities were traditional circumcision is practiced.

Little is known about mechanisms by which HIV gains access to the MGT. While circumcision has been shown to be an effective HIV intervention strategy, 40% of circumcised men are not protected after MMC, and condom usage is still regarded the best HIV prevention method, despite low adherence globally (101–103). The efficiency of MMC indicates that the removal of the foreskin reduces a natural environmental niche for HIV-1 acquisition in the MGT. Due to limited access to penile tissue, there are large gaps of knowledge regarding routes of HIV acquisition in males other than the foreskin. More studies on how HIV-1 has evolved to exploit the MGT architecture – utilizing subclinical inflammation and microbial dysbiosis to its advantage – are required to inform targeted intervention strategies that can prevent acquisition. In addition, a multidisciplinary approach marrying the biology of transmission and acquisition with the identity of sexual networks between MSM and heterosexual populations is a way to understand the HIV-1 epidemic and ways to mitigate transmission.

## Author Contributions

RE, devised and co-wrote the review. AO, co-wrote the review. J-AP, co-wrote the review. HJ, co-wrote the review. RH, co-wrote the review. CG, shaped and co-wrote the review.

## Conflict of Interest Statement

The authors declare that the research was conducted in the absence of any commercial or financial relationships that could be construed as \a potential conflict of interest.
